# Proteinase-Activated Receptor-2 Agonist Activates Anti-Influenza Mechanisms and Modulates IFN**γ**-Induced Antiviral Pathways in Human Neutrophils

**DOI:** 10.1155/2013/879080

**Published:** 2013-09-22

**Authors:** Micha Feld, Victoria Shpacovitch, Christina Ehrhardt, Michaela Fastrich, Tobias Goerge, Stephan Ludwig, Martin Steinhoff

**Affiliations:** ^1^Department of Dermatology, Heinrich-Heine University, 40225 Düsseldorf, Germany; ^2^Leibniz-Institute for Analytical Sciences (ISAS), 44139 Dortmund, Germany; ^3^Institute of Molecular Virology, ZMBE, Westfälische Wilhelms-University of Münster, 48149 Münster, Germany; ^4^Department of Dermatology and Boltzmann Institute for Immunobiology of the Skin, Westfälische Wilhelms-University of Münster, 48149 Münster, Germany; ^5^Departments of Dermatology and Surgery, University of California San Francisco (UCSF), San Francisco, CA 94143, USA

## Abstract

Proteinase-activated receptor-2 (PAR_2_) is expressed by human leukocytes and participates in the development of inflammatory diseases. Recent studies demonstrated an ability of PAR_2_ agonist to enhance IFN**γ**-induced antiviral responses of human leukocytes. However, the precise cellular antiviral defense mechanisms triggered in leukocytes after stimulation with IFN**γ** and/or PAR_2_ agonist remain elusive. Therefore, we aimed to identify neutrophil defense mechanisms involved in antiviral resistance. Here we demonstrated that PAR_2_ agonist enhanced IFN**γ**-related reduction of influenza A virus (IAV) replication in human neutrophils. PAR_2_-mediated decrease in IAV replication was associated with reduced NS-1 transcription. Moreover, PAR_2_-dependent neutrophil activation resulted in enhanced myeloperoxidase degranulation and extracellular myeloperoxidase disrupted IAV. The production of ROS was elevated in response to PAR_2_ activation. Interestingly, IFN**γ** did not influence both effects: PAR_2_ agonist-triggered myeloperoxidase (MPO) release and reactive oxygen species (ROS) production, which are known to limit IAV infections. In contrast, orthomyxovirus resistance gene A (MxA) protein expression was synergistically elevated through PAR_2_ agonist and IFN**γ** in neutrophils. Altogether, these findings emphasize two PAR_2_-controlled antiviral mechanisms that are independent of or modulated by IFN**γ**.

## 1. Introduction

The impact of proteinase-activated receptor-2 (PAR_2_) activation on inflammatory processes varies and depends on the stage of disease and the primary cell type(s) involved in disease progression [1, 2]. Trypsin, tryptase, and pathogen-derived proteases could trigger PAR_2_ activation [3]. However, these enzymes cause PAR_2_-dependent as well as PAR_2_-independent effects [4, 5]. Moreover, trypsin-like serine proteases could assist influenza A replication via cleavage of viral hemagglutinin [6]. Together, these facts exclude the use of trypsin and tryptase as appropiate PAR_2_ activators in studies involving influenza A virus. Thus we used influenza A/FPV/Bratislava/79 (H7N7) containing a multibasic-cleavage site, which efficiently replicates without the necessity of trypsin. Moreover, specific synthetic PAR_2_-activating peptides, used in our study, do not affect hemagglutinin maturation but reportedly serve as important tools for investigating the role of PAR_2_ activation in a wide range of anti-influenza responses.

Interferon-*γ* (IFN*γ*) regulates the cellular antiviral state and shapes the antiviral and inflammatory response [7]. Recent *in vitro* and *in vivo* studies revealed a cooperation between IFN*γ* and PAR_2_ agonists in the induction of antiviral responses and in the regulation of the chemokine levels [8–10]. However, it remains unclear which cellular antiviral defence mechanism(s) in leukocytes are affected after concomitant IFN*γ* and PAR_2_ agonist application.

Neutrophils participate in the defence against influenza A virus (IAV) infection. Although it is well established that neutrophils contribute to lung injury during IAV infection, neutropenia is associated with enhanced virus replication in lungs and high mortality [11]. Moreover, neutrophils limit spreading in the organism of IAV strains with intermediate or high virulence [12]. Human neutrophils express functional PAR_2_ [13, 14], which regulates motility and bactericidal activity of neutrophils [1, 10]. Although the PAR_2_-induced bactericidal activity is not enhanced in the presence of IFN*γ* in neutrophils [10], PAR_2_ agonist and IFN*γ* synergize boosting anti-influenza effects in human monocytes [8]. Nonetheless, the role of PAR_2_ and IFN*γ* in neutrophils during IAV infection remains elusive.

Neutrophils possess a broad spectrum of weapons against viral and microbial pathogens including compounds of neutrophil granules (defensins, elastase, and some others), reactive oxygen species (ROS), and orthomyxovirus resistance gene (Mx) proteins [15, 16]. Thus, we investigated how PAR_2_ activation affects IAV replication in neutrophils and which defence mechanism(s) are activated. We also evaluated whether PAR_2_ agonist and IFN*γ* synergize to strengthen the antiviral response. 

## 2. Material and Methods

### 2.1. Materials

Human PAR_2_-activating peptide with the sequence *trans-cinnamoyl*-LIGRLO-NH_2_ (tcAP) and the reverse peptide with the sequence *trans-cinnamoyl*-OLRGIL-NH_2_ (tcRP) were synthesized at the University of Calgary (Peptide Synthesis Facility, Dr. D. McMaster, Calgary, Canada; http://www.ucalgary.ca/peptides/) and used at a concentration of 10^−4 ^M as described previously [8]. Human recombinant IFN*γ* was received from Peprotech (Hamburg, Germany) and used at a concentration of 200 U/mL. The following antibodies were used: mouse anti-human *β*-actin (Sigma Aldrich); mouse monoclonal anti-MxA antibody (M143) which was a kind gift from the Department of Virology of the University of Freiburg and was used as described previously [17]. All cell culture reagents were obtained from PAA (Cölbe, Germany) or otherwise stated in the text.

### 2.2. Isolation and Culture of Neutrophils

Buffy coats from healthy adult human volunteers were obtained from the Deutsches Rotes Kreuz (Münster, Germany), and neutrophils were prepared as described previously [18]. Isolated neutrophils (1–1.5 × 10^6^ cells/mL) were allowed to recover in RPMI 1640 (Lonza) supplemented with 1% L-glutamine, 1% nonessential amino acids, 1% penicillin/streptomycin, and 0.9% fetal calf serum for at least 1 hr. 

### 2.3. Virus and Infections

Avian influenza virus A/FPV/Bratislava/79 (H7N7; FPV) was originally obtained from the virus strain collection of the Institute of Virology (Justus-Liebig-University, Gießen, Germany). For infection, human neutrophils were washed with PBSi (PBS supplemented with 0.01% CaCl_2_, 0.01% MgCl_2_, and 0.2% bovine serum albumin (BSA)) and infected with a multiplicity of infection of 0.75. Therefore, the virus was diluted accordingly in PBSi and applied to the cells for 30 min at 37°C and 5% CO_2_. Then, the inoculum was aspirated and replaced by RPMI 1640 supplemented with 2 mM L-glutamine, 1% nonessential amino acids, 1% penicillin and streptomycin, 0.2% BSA, 0.01% CaCl_2_, and 0.01% MgCl_2_. For inhibitor studies, 1 mM myeloperoxidase (MPO) inhibitor (Calbiochem) or vehicle was added to the medium. Subsequently, cells were stimulated with agonists or left untreated. Cells were incubated for 0–20 hrs (as indicated in the text) at 37°C and 5% CO_2_ depending on the readout system. In a second experimental approach, neutrophils were primed with agonists for 2 hrs and, subsequently, infected with IAV for 30 min as described above. Following infection primed cells were rechallenged with agonists (b/a stimulation protocol) for 20 hrs. Only if stated in the text, the b/a stimulation protocol was applied. 

### 2.4. Quantification of Neutrophil Degranulation

After recovery, neutrophils were treated for 2 hrs with the indicated agonists or used immediately without prestimulation. Then, cells were spun down and resuspended at a ratio of 1 × 10^6^ cells per 100 *μ*L in PBS. Neutrophils were pretreated with 5 *μ*g/mL of the degranulation-promoting agent Cytochalasin B (Sigma Aldrich) (for 5 min at 37°C) and, subsequently, rechallenged with appropriate agonists for 30 min at 37°C. Cells were removed by centrifugation, and the supernatant was analysed for elastase and MPO activity. To measure the elastase release, the supernatant was prediluted 1/100 and incubated with 100 *μ*g/mL alpha-1-antitrypsin (*α*1AT) for 30 min at 37°C. Then, elastase/*α*1AT mixture was applied to PMN elastase ELISA (Abnova, Heidelberg, Germany). The assay was performed according to the manufacturer's instructions. To quantify the MPO levels, 100 *μ*L of degranulated supernatant was mixed with 100 *μ*L 3,3′,5,5′-tetramethylbenzidine (TMB) liquid substrate (Sigma Aldrich). Changes in the optical density at 630 nm were monitored for 20 min.

### 2.5. IAV Disruption by Neutrophil Supernatant

Supernatant from degranulated neutrophils was prepared as described above. The virus was diluted to 1 × 10^6^ PFU/mL. Then, neutrophil supernatant and virus dilution were mixed in a ratio of 1 : 1 and supplemented with 1 mM H_2_O_2_ (Merck) or vehicle as indicated. After incubation for 1 hr at 37°C and 5% CO_2_, samples were collected and analysed in a standard plaque assay.

### 2.6. Measurement of Intracellular Reactive Oxygen Species (ROS)

Intracellular generation of ROS was detected using the fluorescent dye 5-(and-6)-chloromethyl-2′,7′-dichlorodihydrofluorescein diacetate (CM-H2DCFDA) (Invitrogen). To induce ROS production, neutrophils (1.5 × 10^6^ cells/mL) were stimulated with the indicated agonists in the absence of cytochalasin B. Thirty minutes before the stimulation was stopped, 5 *μ*M CM-H2DCFDA was added. Then, cells were put on ice, spun down at 4°C, and washed with PBS. Finally, neutrophils were resuspended in PBS supplemented with 1% FCS, 2 mM EDTA, and 2% paraformaldehyde and analysed with the FACScalibur and Cell Quest Pro software (BD Biosciences). 

### 2.7. Calcium Mobilization Studies

Changes in intracellular calcium levels were measured as described previously [8, 14, 15]. Briefly, isolated neutrophils were washed, resuspended in HEPES-buffered salt solution (140 mM NaCl, 3 mM KCl, 0.4 mM Na_2_HPO_4_, 10 mM HEPES, 5 mM glucose, and 1 mM MgCl_2_ (pH 7.4)) with or without 0.8 mM CaCl_2_, and incubated with 3.5 *μ*M Fura-2 acetoxymethyl for 30 min at 37°C. Cells were washed twice, resuspended in HEPES-buffered salt solution with or without 0.8 mM CaCl_2_, and PAR_2_-triggered elevation in intracellular calcium levels was measured in a FluoroMaxx spectrophotometer (Yobin Yvon). For inhibitor studies, cells were pretreated with 100 *μ*M 2-aminoethoxydiphenyl borate (2-APB) for 3 min before the PAR_2_ agonist was applied. 

### 2.8. Real-Time RT-PCR

Steady-state levels of MxA, oligoadenylate synthetase (OAS), and the viral nonstructural protein (NS-1/2) were evaluated by real-time fluorescence detection using Absolute SYBR Green ROX mix (Applied Biosystems, Foster City, CA, USA). Reactions in duplicate were analysed in an ABI Prism 7300 sequence detector supplied with SDS 2.1 software (Applied Biosystems). Specific primer pairs were used: MxA forward, 5′-AGAGAAGGTGAGAAGCTGATCC-3′, and reverse, 5′-TTCTTCCAGCTCCTTCTCTCTG-3′; oligoadenylate synthetase (OAS) forward, 5′-GCTCCTACCCTGTGTGTGTGT-3′, and reverse, 5′-TGGTGAGAGGACTGAGGAAGA-3′; NS-1/2 forward, 5′-GAGGACTTGAATGGAATGATAACA-3′, and reverse, 5′-GTCTCACTTCTTCAATCAACCATC-3′.

### 2.9. Immunoblot Analysis

Stimulated neutrophils were collected, disrupted in preheated (100°C) lysing buffer (4 M urea, 0.5 M Tris pH 6.8, 25% glycerine, 10% SDS, and 0.005% bromophenol blue) supplemented with freshly prepared 1x protease inhibitor cocktail (Roche Diagnostics) and 200 mM dithiothreitol, and boiled for 5 min. Whole cell lysate preparations of stimulated neutrophils were separated by SDS-PAGE and transferred onto nitrocellulose membrane. To assess MxA expression 35 *μ*g of protein lysate was applied per lane. Densitometric analysis was performed using ImageJ software.

### 2.10. Statistical Analysis

Results are expressed as mean ± SEM. At least three independent experiments were performed (*n* ≥ 3). Statistical evaluation was done by an analysis of variance and Student's *t-*test or Wilcoxon matched-pairs signed rank test. Significance was set at *P* < 0.05.

## 3. Results

### 3.1. IAV Replication in Neutrophils Is Reduced by PAR_2_ Agonist and IFN*γ*

**Figure 1:**
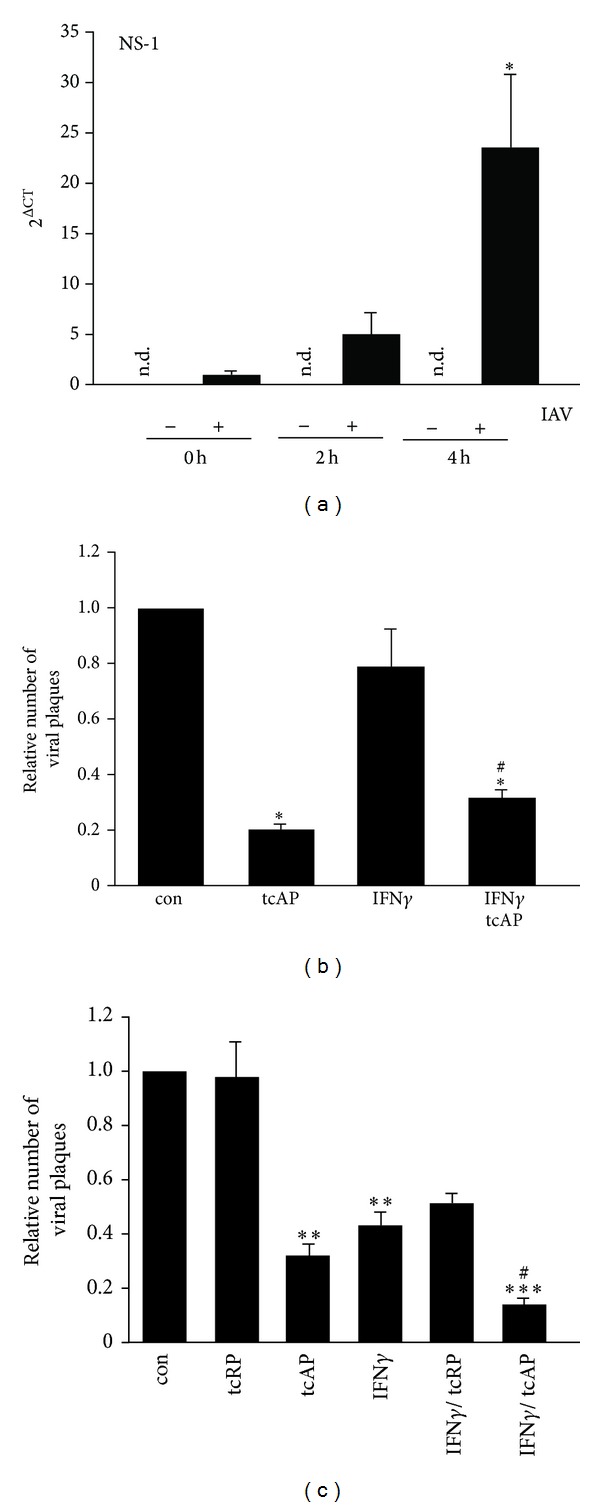
IAV replication in neutrophils was restricted by PAR_2_ activation and IFN*γ*. (a) Replication of IAV in neutrophils was determined by detection of NS-1 mRNA levels at different time points after infection. At 4 hrs, a significant induction of NS-1 mRNA expression was revealed. In noninfected neutrophils NS-1 mRNA was not detectable. (b) IAV-infected neutrophils were treated with agonists as indicated for 20 hrs. Analysis of IAV titers showed a significant reduction in PAR_2_ agonist and PAR_2_ agonist/IFN*γ* treated neutrophils. (c) In cells that were primed with agonists for 2 hrs, infected with IAV for 30 min, and rechallenged with agonists for 20 hrs, both PAR_2_ agonist and IFN*γ* decreased viral replication. Moreover, combining PAR_2_ agonist and IFN*γ* further reduced IAV titers as compared to both agonists alone. For student's *t*-test: ^#,∗^ *P* < 0.05; **P < 0.01; ***P < 0.005. The symbol ∗ marks the significance as compared to control and the symbol # as compared to IFN*γ* sample.

Previously, we revealed that PAR_2_ and IFN*γ* cooperate to interfere with IAV replication in human monocytes [8]. Here, we investigated whether such a cooperation also exists in neutrophils, as they appear to play an important role during IAV infections. Therefore, we aimed to confirm the replication of the avian IAV strain H7N7 in human neutrophils. Indeed, infection of neutrophils led to a time-dependent upregulation of viral NS-1 mRNA after 2 and 4 hrs. In noninfected neutrophils, viral NS-1 mRNA was not detectable (Figure 1(a)). Next, we treated IAV-infected neutrophils with PAR_2_-tcAP, IFN*γ*, or a combination thereof and measured viral titers after 20 hrs. PAR_2_ agonist stimulation decreased IAV titers by 80 ± 2%, whereas IFN*γ* treatment had no significant effect (Figure 1(b)). Concomitant stimulation with PAR_2_ agonist and IFN*γ* reduced IAV progeny by 3-4-fold (Figure 1(b)). To evaluate whether primed neutrophils are more resistant to IAV replication, we primed neutrophils with PAR_2_ agonist, IFN*γ*, or their combination for 2 hrs before cells were infected with IAV and rechallenged cells after infection (b/a-stimulation). In this stimulation protocol, PAR_2_ and IFN*γ* reduced viral titers by 68 ± 4% and by 57 ± 5%, respectively (Figure 1(c)). Combining PAR_2_ agonist and IFN*γ* additively decreased IAV titers by approximately 86 ± 2% (Figure 1(c)). Scrambled PAR_2_ peptide (tcRP) was used as control and did not affect viral titers (Figure 1(c)). Together, our data revealed that IAV replicates in neutrophils and that PAR_2_ agonist and IFN*γ* reduce IAV titers.

### 3.2. PAR_2_ Activation Triggers Degranulation and Production of Reactive Oxygen Species (ROS) in Neutrophils

Myeloperoxidase (MPO) as well as other compounds of azurophil granules were demonstrated to have anti-influenza activity [19, 20] and, thus, may contribute to host protective rather than harmful functions. PAR_2_-AP was shown to increase plasma MPO activity indicating enhanced neutrophil degranulation in mice [21]. Therefore, we analysed whether stimulation with PAR_2_-tcAP or IFN*γ* triggers human neutrophil degranulation of azurophil granules *in vitro*. In our preliminary experiments, where neutrophils (app. 4 × 10^6^ cells/100 *μ*L) were primed with PAR_2_ agonist for 2 hrs, a second dose of PAR_2_ agonist elicited the release of elastase. However, variations in the magnitudes of the effect did not allow this effect of PAR_2_ agonist to reach statistical significance (unpublished observations). 

**Figure 2:**
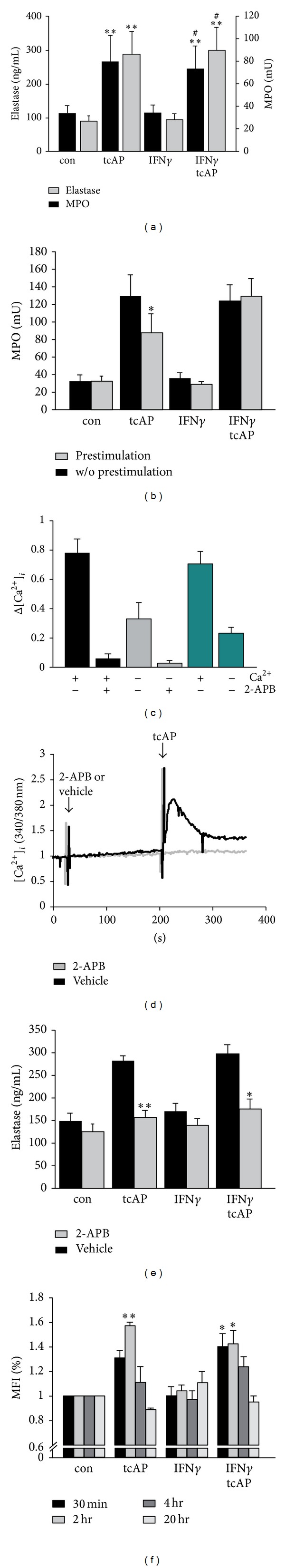
PAR_2_ stimulation induced neutrophil degranulation in a Ca^2+^-dependent manner and upregulated ROS production. Neutrophils were treated as described in Material and Methods Section. (a) After stimulation with PAR_2_ agonist and IFN*γ*, the concentration of MPO and elastase was quantified in cytochalasin B primed neutrophils. (b) Comparing MPO levels in cytochalasin B primed neutrophils that were either pretreated with agonists (b/a-stimulation) or not showed a reduction in PAR_2_ agonist stimulated neutrophils. Concomitant stimulation with PAR_2_-agonist and IFN*γ* induced similar MPO levels in both pretreated and nonpretreated cells. (c, d) Neutrophils were loaded with Fura-2 AM (30 min), washed, and then PAR_2_ agonist was added, and calcium mobilization was investigated. The availability of extracellular Ca^2+^ led to increased intracellular calcium levels after PAR_2_ agonist application. 2-APB almost completely blocked intracellular Ca^2+^ fluxes, independent of extracellular Ca^2+^. (e) Pretreatment of neutrophils with 2-APB prevented PAR_2_ agonist induced elastase release. (f) Changes in ROS levels were measured using a fluorescent substance (CM-H2DCFDA) that was added 30 min before the stimulation was stopped (see Material and Methods Section). Only at early time points, PAR_2_ agonist elevated ROS level as measured by changes of the MFI. IFN*γ* did not induce ROS upregulation. For student's *t*-test: ^#,∗^ *P* < 0.05; **P < 0.01. The symbol ∗ marks the significance as compared to control and the symbol # as compared to IFN*γ* sample.

In contrast, preactivation of neutrophils with cytochalasin B led to a robust elevation of elastase and MPO release after PAR_2_ activation. Basal release of MPO and elastase in cytochalasin B primed neutrophils was determined as 26.9 ± 4.6 mU and 113.6 ± 21.0 ng/mL, respectively (Figure 2(a)). Further addition of PAR_2_-tcAP enhanced extracellular MPO (86.5 ± 19.3 mU) and elastase (265.8 ± 76.4 ng/mL) levels significantly, but degranulation was unaffected by IFN*γ*. Concomitant stimulation with PAR_2_ agonist and IFN*γ* failed to overcome the effect induced by PAR_2_-tcAP alone. 

PAR_2_-tcAP primed, then cytochalasin B treated and rechallenged neutrophils (see “Quantification of Neutrophil degranulation” in Material and Methods Section for details) behaved in different way. Applying the b/a stimulation, the second PAR_2_ activation resulted in significantly less elevated MPO levels (87.9 ± 20.4 mU) as compared to 128.6 ± 24.0 mU in nonpreactivated cells (Figure 2(b)). However, this reduction was not detected in neutrophils activated with both PAR_2_ agonist and IFN*γ* (Figure 2(b)). 

Because degranulation is often triggered by Ca^2+^ signaling, we also investigated the contribution of Ca^2+^ fluxes to PAR_2_-induced degranulation. PAR_2_ agonist induced a rapid increase in intracellular Ca^2+^ signaling in both Ca^2+^-free or Ca^2+^-supplemented buffer. However, extracellular Ca^2+^ boosted PAR_2_ agonist-induced intracellular calcium signals by 3-fold as compared to extracellular Ca^2+^ starvation (Figure 2(c), green columns). However, PAR_2_-induced release of azurophil granules was independent of additional extracellular Ca^2+^ (data not shown). 2-APB is known as an inhibitor of InsP3-induced Ca^2+^ release and, probably, concomitant Ca^2+^ entry [22]. 2-APB inhibited PAR_2_-induced Ca^2+^ release (Figures 2(c) and 2(d)) and, subsequently, reduced degranulation of azurophil granules as measured by elastase release (Figure 2(e)). 

Reactive oxygen species (ROS) shape the inflammatory response during IAV infections [23]. In neutrophils, PAR_2_-tcAP, without any priming with cytochalasin B, induced ROS production that peaked at 2 hrs and then declined to baseline levels within 20 hrs. At 2 hrs, PAR_2_ significantly upregulated ROS levels by 1.6 ± 0.2-fold as compared to controls. However, combination of PAR_2_ agonist and IFN*γ* was not more potent in induction of ROS than PAR_2_-tcAP alone. IFN*γ* alone did not affect ROS production in neutrophils (Figure 2(f)). Together, our data indicated a regulatory role for PAR_2_, but not for IFN*γ*, in neutrophil degranulation of azurophil granules and ROS production.

### 3.3. MPO Activity Disrupts IAV, but MPO Inhibition Is not Sufficient to Reverse PAR_2_ Agonist-Induced Reduction of IAV Replication

**Figure 3:**
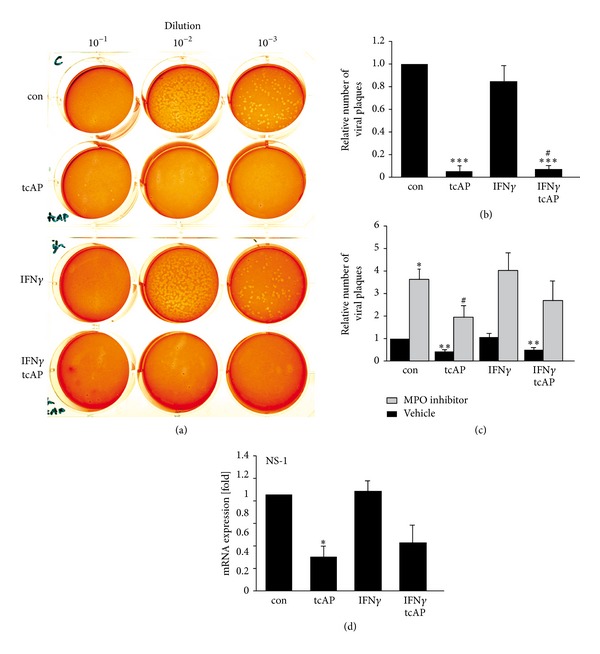
Influenza titers were controlled through extracellular MPO and on transcriptional level through PAR_2_. (a, b) DF from stimulated neutrophils was supplemented with H_2_O_2_, and the virucidal activity was determined. DF from PAR_2_ agonist treated neutrophils disrupted IAV. (c) Application of a MPO inhibitor enhanced viral titers. Interestingly, despite MPO inhibition, PAR_2_ activation reduced viral replication in neutrophils. (d) Analysis of viral gene replication displayed reduced NS-1 mRNA expression in PAR_2_ agonist stimulated neutrophils. IFN*γ* had no effect on viral replication. For student's *t*-test: ^#,∗^ *P* < 0.05; **P < 0.01; ***P < 0.005. The symbol ∗ marks the significance as compared to control and the symbol # as compared to IFN*γ* sample.

MPO and ROS are required for extracellular disruption of IAV [20]. Therefore, we hypothesized that degranulation fluid (DF) from PAR_2_-activated neutrophils may disrupt IAV. Neutrophils were treated with PAR_2_ agonist, IFN*γ*, or their combination, and the DF was collected. In the presence of H_2_O_2_, DF from PAR_2_ agonist-treated neutrophils decreased IAV titers by 20-fold (95 ± 5%) as compared to controls, whereas DF from IFN*γ*-stimulated neutrophils only marginally decreased viral titers by 14 ± 1.5% (Figures 3(a) and 3(b)). DF from PAR_2_ agonist and IFN*γ* costimulated neutrophils In the presence of H_2_O_2_, the DF from PAR2 agonist and IFN*γ* co-stimulated neutrophils reduced viral titers by 20-fold as compared to controls. In the absence of H_2_O_2_, DF did not reduce viral titers (data not shown). Of note, purified elastase failed to disrupt IAV (data not shown).

To further specify the role of MPO and H_2_O_2_ in neutrophil response against IAV, we treated IAV-infected neutrophils with a specific MPO inhibitor prior to stimulation with PAR_2_ agonist, IFN*γ*, or their combination. In IAV-infected untreated neutrophils, MPO inhibition increased viral titers by approximately 4-fold (Figure 3(c)). It is worth to notice that PAR_2_ activation significantly decreased viral titers 2-fold (50 ± 10%) even in the presence of the MPO inhibitor (Figure 3(c)). In contrast, IFN*γ* did not reduce viral titers in neutrophils treated with MPO inhibitor. The combination of PAR_2_-tcAP and IFN*γ* showed a trend to decrease viral progeny even in the absence of functional MPO.

We next analysed whether reduction of viral progeny originated from intracellular events. Therefore, neutrophils were infected with IAV. Further, viral NS-1 mRNA synthesis was measured as a marker for virus replication. In the case of PAR_2_ agonist as well as combined PAR_2_ agonist and/IFN*γ* costimulation viral NS-1 mRNA levels were decreased by 70 ± 10% and 50 ± 18%, respectively (Figure 3(d)). Again, IFN*γ* alone had no effect on reduction of viral NS-1 mRNA synthesis (Figure 3(d)).

Thus, PAR_2_ agonist-induced disruption of IAV is associated with the MPO-H_2_O_2_ axis and intracellular antiviral mechanisms interfering with IAV gene transcription, indicating at least two PAR_2_-regulated antiviral mechanisms.

### 3.4. PAR_2_ Agonist Stimulation Affects IFN*γ*-Induced MxA Expression in Human Neutrophils

**Figure 4:**
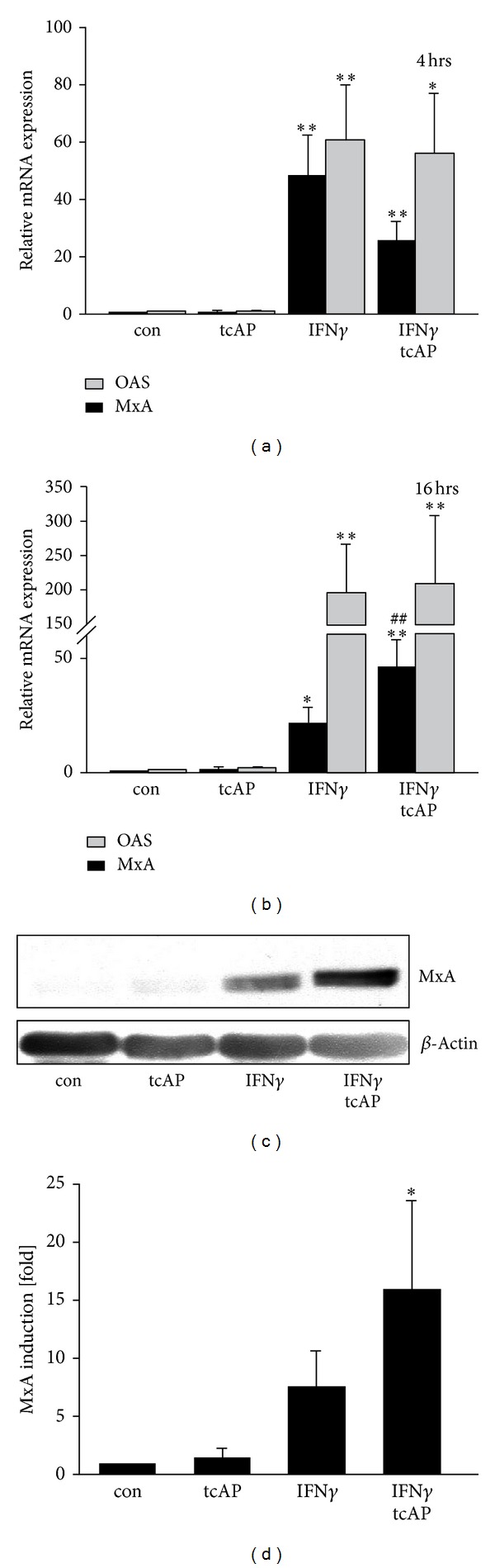
Regulation of MxA and OAS expression. (a, b) IFN*γ*-induced expression of OAS remained unaffected after application of PAR_2_ agonist. But PAR_2_ agonist synergizes with IFN*γ* to elevate MxA expression at 16 hrs, although this effect was not evident at early time points (4 hrs). (c, d) MxA expression was further analysed on protein level. Similar to mRNA results, concomitant stimulation with PAR_2_ agonists and IFN*γ* induced MxA protein (at 20 hrs time point). In contrast, IFN*γ* upregulated MxA only slightly and nonsignificantly. For students *t*-test: ^#,∗^ *P* < 0.05; **P < 0.01. The symbol ∗ marks the significance as compared to control and the symbol # as compared to IFN*γ* sample. (d) Densitometric results were received for Western blot samples. Wilcoxon matched-pair signed rank test was applied for analysis: *P < 0.05 as compared to control.

We investigated the regulation of OAS and MxA levels. IFN*γ* triggered OAS mRNA expression at 4 hrs and 16 hrs by 61 ± 18-fold and 197 ± 88-fold, respectively, as compared to controls (Figures 4(a) and 4(b)). When applied together, PAR_2_ agonist and IFN*γ* induced OAS mRNA expression at 4 hrs and 16 hrs by 56 ± 20-fold and 210 ± 96-fold, respectively (Figures 4(a) and 4(b)). PAR_2_ agonist alone did not induce either OAS or MxA expression (Figures 4(a) and 4(b)). IFN*γ* induced MxA mRNA levels by 48 ± 13-fold (4 hrs) and 20 ± 7-fold (16 hrs) as compared to controls. Concomitant stimulation with PAR_2_ agonist and IFN*γ* enhanced MxA expression by 25 ± 6-fold at 4 hrs and 46 ± 11-fold at 16 hrs (Figures 4(a) and 4(b)) as compared to controls. Since mRNA upregulation not necessarily leads to protein upregulation, the mRNA data were further verified by analysis of MxA on protein levels. As shown in Figures 4(c) and 4(d), the analysis of MxA protein expression after agonist stimulation resembled the expression profile observed on mRNA level. However, only the concomitant stimulation with PAR_2_ agonist and IFN*γ* upregulated the MxA protein expression significantly (Figures 4(c) and 4(d)). Although MxA was also slightly increased after IFN*γ* treatment alone, this effect never reached statistical significance. In two samples out of six, MxA was just barely detectable after IFN*γ* stimulation (data not shown). However, in other samples MxA expression was detectable and just slightly enhanced after IFN*γ* stimulation (Figures 4(c) and 4(d)). 

Thus, PAR_2_ agonist stimulation appears to be an important factor enhancing IFN*γ*-induced expression of MxA. 

## 4. Discussion

The central hypothesis of our current work focuses on the role of PAR_2_-mediated degranulation-dependent antiviral responses and PAR_2_-induced intracellular defence mechanisms. Therefore, we investigated whether PAR_2_ activates MPO release or triggers intracellular events that interfere with transcription of viral genes. We also explored whether antiviral defence mechanisms (e.g., MxA) might be regulated by PAR_2_ agonist and IFN*γ*. 

First of all, we proved the ability of PAR_2_ and IFN*γ* to synergize reducing IAV replication in human neutrophils (Figure 1). Indeed, simultaneous pretreatment with both agonists followed by their coapplication after infection was more effective in the reduction of IAV replication than any of agonists alone (Figure 1(c)). Moreover, PAR_2_ agonist application, but not IFN*γ*, reduced IAV amplification in infected human neutrophils even without pretreatment (Figure 1(b)), suggesting different antiviral activities of IFN*γ* and PAR_2_ agonist. We hypothesized that PAR_2_ elicits immediate effects based on neutrophil degranulation, whereas the antiviral action of IFN*γ* is time-delayed. Thus, further, we investigated cellular anti-influenza defence mechanisms triggered by both substances. 

Neutrophilic MPO was shown to possess anti-pathogenic activity in the presence of H_2_O_2_ [20]. Moreover, PAR_2_-AP application was demonstrated to enhance MPO release in mice [21]. However, it remained unclear whether PAR_2_ agonists directly induce neutrophil degranulation and whether released MPO inactivates or disrupts the IAV strain H7N7. We revealed that PAR_2_ agonist application triggers Ca^2+^-dependent degranulation of human neutrophils and, thus, enhances MPO and elastase release (Figure 2). To measure degranulation, we pretreated neutrophils with cytochalasin B. Cytochalasin B is an artificial substance, which mimics neutrophil priming potentially via induction of a state of GPCRs reactivation [24]. However, in preliminary studies, rechallenge of PAR_2_ agonist-primed neutrophils also showed a trend of elevated elastase levels indicating that degranulation may partially occur without cytochalasin B pretreatment (unpublished data). Interestingly, PAR_2_ agonist stimulation, without cytochalasin B pretreatment, was capable of enhancing ROS production by human neutrophils (Figure 2(f)), amongst which H_2_O_2_ is the substrate for MPO. Moreover, we demonstrated that DF derived from PAR_2_ agonist-activated neutrophils contains MPO and disrupts extracellular IAV (Figure 3(a)), indicating a MPO-dependent anti-influenza action. In contrast, IFN*γ* failed to enhance PAR_2_-triggered MPO release, and ROS production (Figure 2). Thus, PAR_2_ appears to induce an anti-influenza defence mechanism in human neutrophils based on degranulation, MPO release and ROS production. However, these mechanisms are clearly independent of and not regulated by IFN*γ* and, thus, represent no cross-point regarding simultaneous PAR_2_ and IFN*γ* antiviral action. 

Although we demonstrated a substantial role for MPO in influenza disruption (Figure 3(a)), application of a MPO inhibitor did not completely reverse the downregulation of intracellular IAV replication in PAR_2_ agonist-activated neutrophils (Figures 3(b) and 3(c)), suggesting the existence of a redundant mechanism(s) that are controlled by PAR_2_. For example, the defensin, cathelicidin LL37, which is stored in neutrophil secondary granules, has been shown to exert anti-influenza activity [25]. Moreover, PAR_2_ agonist application also reduced NS-1 production in IAV infected neutrophils (Figure 3(d)), further pointing to PAR_2_-mediated transcriptional regulation during virus replication. 

IFN*γ* application as a pretreatment and during infection (b/a stimulation) was able to reduce IAV replication in human neutrophils (Figure 1(c)). Moreover, in the b/a stimulation model, concomitant IFN*γ* and PAR_2_ stimulation reduced IAV amplification in human neutrophils as compared to other stimulations (Figure 1(c)). Thus, antiviral mechanisms might require the presence of both PAR_2_ agonist and IFN*γ*. Indeed, application of PAR_2_ agonist together with IFN*γ* resulted in stronger induction of MxA mRNA expression as compared to the stimulation with IFN*γ* alone (Figure 4(b)). Antiviral MxA, classically inducible by type I interferons [26], was demonstrated to be elevated by IFN*γ* on transcriptional level [27]. To our knowledge, the detection of MxA protein upon IFN*γ* stimulation remains elusive. Although we confirmed the induction of MxA mRNA upon IFN*γ* treatment, we found variations in the MxA protein expression amongst the investigated donors. These variations could not be explained by the Western blot artefacts since the experimental protocol was kept constant during all the time. Only combined PAR_2_ agonist/IFN*γ* stimulation significantly raised MxA protein levels in all investigated samples revealing a potential backup system for type I interferons for efficient fight against IAV infections intracellularly. 2′-5′ oligoadenylate synthetase (OAS) also participates in cellular defence against RNA viruses and could be induced by IFN*γ* [26, 28]. But OAS expression was not affected by PAR_2_ agonist application even in combination with IFN*γ* (Figures 4(a) and 4(b)). Our data suggests that PAR_2_ shapes the antiviral response through activation of a defined set of defence mechanisms. 

In summary, our data demonstrate that PAR_2_ agonist and IFN*γ* synergize to reduce IAV progeny in human neutrophils. Enhanced MxA production is revealed as a cellular antiviral mechanism, which is synergistically activated by PAR_2_ agonist and IFN*γ* in human neutrophils. However, in neutrophils PAR_2_ agonist controls IFN*γ*-independent antiviral mechanism(s) such as enhanced MPO release, ROS production, and reduction of viral gene transcription.

## Abbreviations

2-APB: 2-Aminoethoxydiphenyl borate
*α*1AT: Alpha-1-antitrypsin
AP: Activating peptide
b/a: Before/after
DF: Degranulated fluid
IAV: Influenza A virus
IFN*γ*: Interferon gamma
MPO: Myeloperoxidase
mU: Milli units
MxA: Orthomyxovirus resistance protein A
OAS: Oligoadenylate synthetase
PAR_2_: Proteinase-activated receptor-2
ROS: Reactive oxygen species
RP: Reverse peptide
tc: *Trans-cinnamoyl*.
